# Improving Medical Photography in a Level 1 Trauma Center by Implementing a Specialized Smartphone-Based App in Comparison to the Usage of Digital Cameras: Prospective Panel Study

**DOI:** 10.2196/47572

**Published:** 2024-01-25

**Authors:** Jan Siad El Barbari, Maxim Fikuart, Nils Beisemann, Michael Müller, Hannah Syrek, Paul Alfred Grützner, Jochen Franke, Sven Yves Vetter

**Affiliations:** 1 Department of Orthopaedics and Traumatology BG Klinik Ludwigshafen Ludwigshafen am Rhein Germany; 2 mbits imaging GmbH Heidelberg Germany; 3 Department of Orthopaedics and Traumatology Tauernklinikum Zell am See Austria

**Keywords:** app, device usability, digital camera, medical photo, medical photography, mRay app, PACS, patient care, patient education, picture archiving and communication system, questionnaire, smartphone

## Abstract

**Background:**

Medical photography plays a pivotal role in modern health care, serving multiple purposes ranging from patient care to medical documentation and education. Specifically, it aids in wound management, surgical planning, and medical training. While digital cameras have traditionally been used, smartphones equipped with specialized apps present an intriguing alternative. Smartphones offer several advantages, including increased usability and efficiency and the capability to uphold medicolegal standards more effectively and consistently.

**Objective:**

This study aims to assess whether implementing a specialized smartphone app could lead to more frequent and efficient use of medical photography.

**Methods:**

We carried out this study as a comprehensive single-center panel investigation at a level 1 trauma center, encompassing various settings including the emergency department, operating theaters, and surgical wards, over a 6-month period from June to November 2020. Using weekly questionnaires, health care providers were asked about their experiences and preferences with using both digital cameras and smartphones equipped with a specialized medical photography app. Parameters such as the frequency of use, time taken for image upload, and general usability were assessed.

**Results:**

A total of 65 questionnaires were assessed for digital camera use and 68 for smartphone use. Usage increased significantly by 5.4 (SD 1.9) times per week (95% CI 1.7-9.2; *P*=.005) when the smartphone was used. The time it took to upload pictures to the clinical picture and archiving system was significantly shorter for the app (mean 1.8, SD 1.2 min) than for the camera (mean 14.9, SD 24.0 h; *P*<.001). Smartphone usage also outperformed the digital camera in terms of technical failure (4.4% vs 9.7%; *P*=.04) and for the technical process of archiving (*P*<.001) pictures to the picture archiving and communication system (PACS) and display images (*P*<.001) from it. No difference was found in regard to the photographer’s intent (*P*=.31) or reasoning (*P*=.94) behind the pictures. Additionally, the study highlighted that potential concerns regarding data security and patient confidentiality were also better addressed through the smartphone app, given its encryption capabilities and password protection.

**Conclusions:**

Specialized smartphone apps provide a secure, rapid, and user-friendly platform for medical photography, showing significant advantages over traditional digital cameras. This study supports the notion that these apps not only have the potential to improve patient care, particularly in the realm of wound management, but also offer substantial medicolegal and economic benefits. Future research should focus on additional aspects such as patient comfort and preference, image resolution, and the quality of photographs, as well as seek to corroborate these findings through a larger sample size.

## Introduction

Medical photography serves 3 primary purposes: documentation of diseases and procedures, education of patients and medical personnel, and publications in various forms [1-3].

The potential of medical photography lies in its ability to objectify conditions that cannot be properly illustrated by laboratory results or medical imaging. This mitigates the risk of biased descriptions or inconsistent measurements across clinicians, particularly those from different specialties [4]. Additionally, unlike written diagnoses, photos can also be proof of missed diagnoses or negative findings, as they are not limited to the perception and experience of the examiner [5].

Additionally, medical photography provides several key advantages, including supporting medical diagnoses in legal cases, enhancing diagnostic accuracy and therapeutic outcomes, improving the quality of consultations, and offering documentation for billing purposes [6-10].

The digital era and the technological revolution with digital imaging and smart devices have further lowered the threshold of medical photography [11-13]. Now, every adequately instructed person can produce medical photos anywhere at any time, repealing restrictions such as the availability of a trained medical photographer, time pressure in an emergency setting, or missing equipment. An exemption from this are specialized photographs for scientific or educational purposes, or in certain kinds of fields, that is, aesthetic surgeries, in which higher resolution and quality necessitate the use of more professional equipment.

However, data security and patient confidentiality need to be upheld. Thus, current guidelines, such as Recital 26 of the European Directive (EU) 2016/679, demand informed consent of the patient; a defined medical need for this photography; correct documentation; and safe, restricted, and password-protected storage with an access log [14].

Nonetheless, in a recent systematic review analyzing ethical aspects of medical photography, the consent process was found to be insufficient or inadequate in 95% of all cases [15].

Digital cameras are mainly used for medical photography in the clinical setting, and most patients seem to prefer these over smartphones [1,11,16]. This is because it is not clear how the data are stored and protected on either a clinically owned or private device. In both cases, people tend to estimate a higher risk of data-protection infringement in smartphones than in cameras, impairing their general acceptance as a reliable tool for medical photography [17-19]. Additionally, patients’ will to approve is influenced not only by individual consent depending on the reasoning, particularly concerning web-based publication, but also by situational preferences, such as the difference between emergency departments and aesthetic surgeries [15]. In high-paced emergency settings such as trauma units, obtaining immediate verbal consent, witnessed by another health care provider, can often be the most practical approach. This should be followed up with written consent as soon as the patient is stabilized or conscious. In contrast, nonemergency cases allow for a more thorough process where the patient can take the time to understand the implications before giving written consent. Across both scenarios, the ethical principles of autonomy, beneficence, and confidentiality remain paramount, ensuring that patient data are secure and used only for medical purposes.

Inherently, both devices bear the same risk of data infringement. Digital cameras cannot be password protected, do not encode their data, and are not usually stored as would be required: either under supervision or locked away. The last aspect is not a problem with smartphones since they are usually kept within reach all the time. Yet if the phone is not password protected or the pictures are saved in the photo app, they can be accessed by people close to the owner or may accidentally be transferred to cloud storage that is not properly protected and where access is not documented [17].

However, if the photos are taken with a password-protected app and are not stored on the device but directly in the picture archiving and communication system (PACS), data protection would be secured. Moreover, this would diminish the risk of false identification of the photo, and so all legislative demands would be met.

The use of smartphones with apps that fulfill the data-protection requirements in medical photography is being increasingly examined. Yet an extensive literature search revealed that no study has compared the use of such an app with digital cameras in terms of the quantity and efficiency of medical photography [1,3,16-18,20-22].

The following hypothesis was tested: using an app for medical photography would increase the quantity of pictures taken and the efficiency of this process.

## Methods

### Study Design

A prospective panel study design with 3 stages was chosen. This was realized in the period from June to November 2020 at a level 1 trauma center. No restrictions were made on where and how pictures should be taken, as the usage in clinical routine was to be evaluated. Pictures could be taken in the emergency department, as well as during surgical procedures, in the surgical ward, or in the outpatient clinic. The study focused on general usage patterns and did not collect data on the specific clinical situations in which the photographs were taken or the type of photographs captured. As a first step, the use of digital cameras was assessed using a printed questionnaire, which was handed out to trauma and orthopedic residents of different years of training with the instruction to fill one out at the end of every week. As a second step, this process was repeated after the installation of a specialized medical imaging app on the clinically owned smartphones, using the same questions adapted to smartphone use. At the end of this second stage, a separate web-based questionnaire about the experiences with the app and its usability and interface could be filled out by all members of the medical staff, not just the residents participating in steps 1 and 2. Since the questionnaire was designed to assess what opportunities and benefits could result from the implementation of the smartphone app in comparison to the digital camera, no respective questionnaire for the usage of the camera was deemed necessary. Both questionnaires were specifically designed for this study; an example of each is included as Multimedia Appendices 1-3.

We used a Likert scale (ranging from –2 to +2) to express experiences with smartphone usage, with –2 representing “strongly nonbeneficial,” +2 as “highly beneficial,” and 0 resembling indifference or no benefit, allowing an intuitive interpretation of the results.

### Digital Camera

Every resident who entered employment received a digital camera (Lumix DMC-FT30, 16 megapixels; Panasonic Corporation) to be used for medical photography. After taking a photo, the resident had to use 1 of 3 workstations in the clinic that offer the capability to upload the photos to the clinical PACS with certain predefined keywords, that is, preoperative or surgical site, to categorize what kind of image had been taken. After uploading, the images had to be deleted from the secure digital card. The digital camera has a 28-mm^2^ sensor with a pixel pitch of 1.3 µm and a resolution of 16 megapixels.

### Specialized Smartphone Application

For clinical communication, each resident received a smartphone (Galaxy xCover 4; Samsung) that only allowed phone calls and viewing of radiological images through mRay (version 6.0.3; mbits imaging GmbH), which is a certified app for medical imaging and processing. The smartphone camera has a 20-mm^2^ sensor with a pixel pitch of 1.1 µm and a resolution of 16 megapixels.

**Figure 1:**
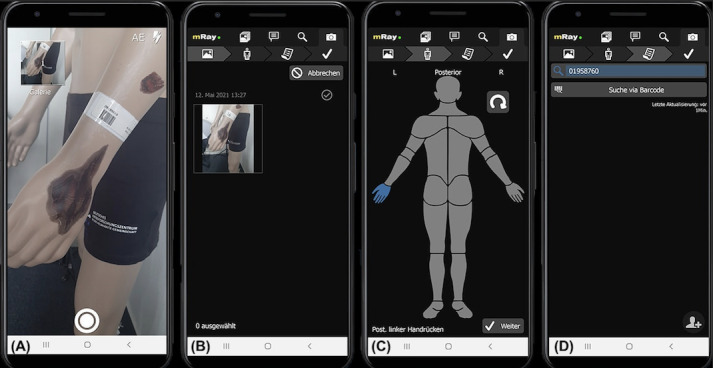
Digital photo documentation workflow through mRay.

In this study, the fully digitalized photo documentation of mRay was used. This is divided into 3 main steps (Figure 1). First, an existing wound is photographed using the smartphone camera (Figure 1A and B). More than 1 picture can be taken if necessary for the patient’s case. In the next step, the wound image can be assigned to the respective body area (Figure 1C). Using a barcode scan or direct search in the clinical PACS, the images can be keyworded and assigned directly to the associated patient’s data (Figure 1D).

### Patient Confidentiality and Data Protection

In accordance with the hospital’s standard operating procedures (Multimedia Appendix 4), each patient is informed upon admission that, in addition to radiological images, clinical photographs necessary for their treatment may also be taken during their course of stay, and a written consent is signed. Additionally, as soon as a photo is to be taken, the patient is educated again about what kind of picture will be taken, where it will be stored, and why it is necessary, and verbal consent is obtained. In emergency situations, another staff member acts as a witness during the process of taking the photograph. Patient consent is subsequently obtained as soon as the individual regains responsiveness.

For digital cameras, data protection protocols require staff to promptly upload images to the clinical PACS, associating them with the respective patient’s file. Once uploaded, images must be deleted from the secure digital card. When not monitored, the camera should be securely stored. Regarding smartphones, they are designated solely for clinical use and feature password protection. Additionally, photographs are exclusively taken through a specialized app, which is also password protected, ensuring direct storage of clinical photographs in the PACS.

### Statistics

Primary end points were effective usage of a camera or smartphone in times per week and the time taken from capturing to uploading the taken pictures in minutes. Secondary end points were the estimated time necessary to archive and display images from the PACS, as well as the intention and reasoning behind the photographs. Additionally, it evaluated how users experienced the introduction and usage of the app, but this was not statistically analyzed. The continuous variables, usage and time to upload, were expressed using mean (SD), and time to archive photos and display them from the PACS were expressed using median (IQR). Evaluations were conducted using the Mann-Whitney *U* test, as these variables were considered estimations despite being interval-scaled as an International System of Units variable. The other categorical variables were analyzed using the chi-square test. The level for statistical significance was set at *P*<.05. Statistics were made using Prism (version 8.2.1; GraphPad Software).

### Ethical Considerations

All procedures performed in this study involving human participants were in accordance with the ethical standards of the Ethics Committee of the State Medical Association of the Rhineland-Palatinate and with the 1964 Declaration of Helsinki and its later amendments or comparable ethical standards. Since the actual photographs taken were acquired as part of the daily clinical routine and were not part of this study, neither informed nor written consent from the patients was necessary. Informed consent was obtained from all individual participants included in the study, and all data were deidentified. No financial compensation was provided to any of the study participants. Data collection, coding, routing, and analysis were in accordance with the legal data protection policy.

## Results

A total of 65 questionnaires regarding digital camera use were collected from June to July 2020, and 68 questionnaires regarding the smartphone app were collected from September to November 2020. The questionnaires were filled out by 5 orthopedic residents. Additionally, 19 fully completed web-based questionnaires were received.

A comparison of the usage of both devices revealed no significant differences. Cameras were used 16.4 (SD 7.7) times per week for taking pictures and 11.2 (SD 9.7) times per week for showing pictures for consultation, whereas for smartphones, these values were 18.8 (SD 5.9; *P*=.10) times per week and 9.8 (SD 4.4; *P*=.47) times per week, respectively. In 17.5% (SD 16.1%) of cases for taking pictures and 18.6% (SD 22.6%) for showing pictures, a missing digital camera was mentioned; however, this issue never arose with smartphones. Technical failure occurred significantly less if the smartphone was used, with a rate of 9.7% (SD 18.2%) of cases with the digital camera and 4.4% (SD 9.1%) with the smartphone (*P*=.04). If the total amount of usage (taking photos and demonstrating them) is adjusted for the cases of missing devices and technical failure, then the corrected usage for the digital camera is 20.8 (SD 11.4) times per week and for the smartphone, 26.2 (SD 10.1) times per week. This difference was statistically significant (*P*=.005; Table 1).

**Table 1:** Primary end points. Values are presented as mean (SD), and *P* values were calculated using the Mann-Whitney U test.

Primary end points		Camera (n=65), mean (SD)	Smartphone (n=68), mean (SD)	*P* value
Usage, adjusted total (times per week)		20.8 (11.4)	26.2 (10.1)	.005
Time to upload (hours or minutes)		14.9 (24.0)^a^	1.8 (1.2)^b^	<.001
**Usage (times per week)**				
	Taking images	16.4 (7.7)	18.8 (5.9)	.10
	Displaying images	11.2 (9.7)	9.8 (4.4)	.47
**Missing device (% of usage)**				
	Taking images	17.5 (16.1)	0 (0)	N/A^c^
	Displaying images	18.6 (22.6)	0 (0)	N/A
Technical failure (% of usage)		9.7 (18.2)	4.4 (9.1)	.04

^a^Hours.

^b^Minutes.

^c^N/A: not applicable.

Statistical differences were also found for the time taken from taking pictures until completion of the upload, the time the technical upload took, and the amount of time needed to view pictures after request (all *P*<.001; Table 1). The time until upload presented the biggest difference, with a mean time of 14.9 (SD 24.0) hours with the digital camera compared to 1.8 (SD 1.2) minutes with the smartphone (Table 1).

A comparison of the time the technical archiving and display of pictures took revealed a significant difference in favor of the smartphone (both *P*<.001, Tables 2 and 3).

**Table 2:** Secondary end points.

Secondary end points		Camera (n=65), n (%)	Smartphone (n=68), n (%)	*P* value	
**Intention^a^**					.31
	Soft tissue conditions	48 (74)	53 (78)		
	Wounds	53 (82)	56 (82)		
	Deformities	19 (29)	22 (32)		
	Range of motion	2 (3)	9 (13)		
	Others	19 (29)	15 (22)		
**Reasoning^a^**					.94
	Legal requirement	34 (52)	32 (47)		
	Improving therapy	17 (26)	21 (31)		
	Preoperative planning	33 (51)	42 (62)		
	Postoperative control	6 (9)	9 (13)		
	Consultation	29 (45)	31 (46)		
	Others	14 (22)	15 (22)		

^a^Percentages exceed 100% because multiple selections were allowed, and the *P* value was calculated using the chi-square test.

**Table 3:** Comparison of time to upload, time to view, and reasons for delay.

Comparison		Camera (n=65), n (%)				Smartphone (n=68), n (%)				*P* value				
		Upload		Viewing		Upload		Viewing		Upload			Viewing	
**Time taken**											<.001			<.001
	<10 s	0 (0)	0 (0)		0 (0)		3 (4)							
	10-30 s	1 (2)	0 (0)		8 (12)		27 (40)							
	30-60 s	4 (5)	11 (17)		27 (40)		14 (21)							
	1-5 min	35 (53)	24 (37)		32 (47)		24 (35)							
	>5 min	26 (40)	30 (46)		1 (1)		0 (0)							
**Reasons for delay^a^**											<.001			<.001
	Technical issues	4 (6)	8 (12)		11 (16)		11 (16)							
	Distance to workstation	49 (75)	37 (57)		0 (0)		0 (0)							
	Organizational reasons	42 (65)	29 (45)		1 (1)		2 (3)							
	None	7 (11)	20 (31)		44 (65)		38 (56)							
	Others	0 (0)	15 (23)		16 (24)		17 (25)							

^a^Percentages exceed 100% because multiple selections were allowed, and the *P* value was calculated using the chi-square test.

However, both groups did differ in the reasons as to why there had been a delay (*P*<.001). The main reasons mentioned with the digital camera were the distance to one of the workstations and organizational reasons, that is, being preoccupied in the operating theater. With the smartphone, there were mostly no reasons for a delay, yet in a quarter (16/68, 24% and 17/68, 25%) of cases, a time lag or app crashes were mentioned (Table 3).

No difference was found in the intention behind the photo, which was mostly documentation of soft tissue conditions (74% and 78%, respectively) and wounds (both 82%; *P*=.31), nor in the reasoning why the photo had been taken (legal requirements, improving therapy, and consultation; *P*=.94).

**Figure 2:**
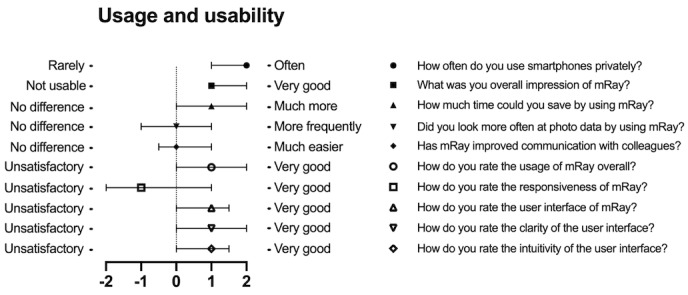
Results of the web-based questionnaire.

The smartphone app’s high acceptance and approval could be deduced from the web-based questionnaire, especially in terms of time savings and an easier workflow (Table 4), with a mostly positive rating on the applied Likert scale (ranging from –2 to 2; Figure 2). There were 2 indifferent evaluations regarding higher usage and improved communication. Only the responsiveness of the app was evaluated negatively with a median of –1, which concurs with the written answers about the occurring time lag and crashes of the app (Table 3 and Figure 2).

**Table 4:** Web-based questionnaire.

Question		Value (n=19), n (%)^a^
**Where do you see the main benefit of mRay in an inpatient trauma setting?**		
	Time savings	17 (90)
	Easier communication with increased quality of treatment	8 (42)
	Easier and more comfortable workflow	13 (68)
	No benefit	0 (0)
**Where the functions of mRay sufficient for carrying out image evaluation?**		
	Yes	18 (95)
	No	1 (5)
**What other functions would you like to see in mRay?**		
	Automated wound measurement	9 (47)
	Automated assessment of wound conditions	5 (26)
	Entering comments on photo findings	11 (58)
	Others	3 (16)

^a^Percentages may exceed 100% because multiple selections were allowed.

## Discussion

### Overview

This prospective study highlighted the advantages of workflow and data security for medical photography by integrating a smartphone app. One key indication is the reduced time taken from capturing the photo to its storage in the PACS: almost instantaneous with smartphones, in contrast to an average 12-hour duration using digital cameras. While viewing photos is feasible at all workstations, uploading is confined to specific stations due to network security concerns. Especially in a time-critical specialty such as traumatology and emergency medicine, such a tool could be particularly beneficial. By lowering the threshold and simplifying the cumbersome workflows of medical photography, the photography process and the number of photos taken could be increased to the benefit of the patient. This would take away the argument that there is no structured assessment or procedure for documentation of acute wounds because the required effort is considered too high and time-consuming [13,23,24].

Such reasoning might originate from studies such as Bronsard et al [23], which tried to establish a pathway for the secure handling of patient data. In their workflow, the photo was sent to a coordinator after it had been taken. This person assessed it and converted it to a DICOM file, which was then cropped by a secretary and finally uploaded. Each week, it took 3 individuals 1-2 hours to generate 1 image from 3-5 photos. It is therefore hardly surprising that they only managed to produce 300 images in 2 years.

Furthermore, previous studies showed that adequate medical photography can improve the care for and decision-making about complex injuries, especially when soft tissues are at risk [7-9,25]. In the case of traumatology, this would mainly be open fractures. Inaccuracies in the description and extent of the concomitant soft tissue injury could affect the planning of the surgical approach and, in the worst case, necessitate the recurrent removal of the dressings to reassess the wound, facilitating a rise in infection risk [26].

The need for an easier and safer way to perform medical photography in traumatology is also enhanced by the argument of Friesen et al [27]. With the rising incidence of older patients in trauma wards, they estimated that 25% of in-house patients could exhibit chronic wounds requiring structured care and documentation [27].

The key aspect that needs to be addressed is the medicolegal aspect and risk of data infringement, which have been shown to be insufficiently addressed in most studies focusing on the adequacy of protocols for patient consent and publication in current practice [15]. First, because the workflow must uphold data security and patient confidentiality, and second, because the acceptance of photography performed with smartphones still needs to be increased, particularly among older patients [18,20].

Anonymous interviews from O’Farrell and Ferreira [1] showed that in 74% of cases, photos taken using a smartphone were not deleted. Furthermore, 58% were stored on a laptop and 26% on a flash drive, while 16% admitted that the device in question was not password protected, and in 21% of cases, third parties could have accessed the pictures. The distribution methods further raise concerns: 58% of the photos were sent for consultation through WhatsApp and 80% through email. Given European Union regulations, these findings underscore a pressing need to address security and privacy challenges in medical photography [1,14].

On the contrary, although the acceptance of clinically owned cameras is fairly high, ranging from 75% to 95%, they also pose a considerable data breach risk. They cannot be password protected, the data are not encoded, and they are mostly not stored safely when not in use [1,17,18,20]. Using an app akin to the one examined in this study, these concerns can be dismissed. Furthermore, the study of Accetta et al [20] showed that in such cases, smartphone photography, under the premise of special information, could reach a comparable acceptance rate of 88%.

Requirements for performing medical photography are an easy and fast appliance, secure storage of data, and prevention of data infringement. This can be achieved using specialized smartphone apps [28].

In addition to the medical value, efficient and extensive medical imaging can also provide economic benefits. If the pictures taken lead to a new diagnosis or therapeutic purpose, then this could be a billable service [2,21,29]. In a study examining smartphone-based medical photography, Jordan et al [21] demonstrated that in 20% of medical audit cases where photos were used, they helped confirm a diagnosis or procedure. This resulted in additional revenue of US $330 per case, amounting to a total of approximately US $70,000 annually.

Besides the possible benefits for acute fracture care and inpatient management, the third benefit could lie in the effect on medical certificates [2,30-33]. These aim to offer an objective assessment of medical outcomes after injuries and rely, therefore, on measurable findings and reliable tools to avoid bias and achieve interrater agreement. Using goniometry to clinically examine the range of motion is important in this regard, but the interrater reliability and agreement for this are not remarkably high. However, Naylor et al [32] could prove that measuring the range of motion from photos taken could achieve an agreement rate of >0.983.

Finally, a key aspect of modern medicine is the informed consent of patients and patient education, as patient compliance and outcomes could be beneficially affected by this [1,34]. Nair et al [22] showed that over two-thirds of patients stated that after being shown images of their condition, their understanding of their condition increased, they believed that this had improved their therapy, and they would therefore recommend this approach to other patients.

In the future, additional applications, such as automatic measurement and categorization of wounds, could be possible if standardized acquisition of these photos can be achieved [35-38]. This could be further simplified if technology such as light detection and ranging scanners becomes widely available on smartphones. Then maintaining specific distances or including measurement references would no longer be necessary for accurate measurements, especially in depth [38-40].

### Limitations

This study has some limitations. Despite its prospective design, the sample size is quite small, and so the evidence base is limited. Additionally, the study was restricted to a single study site. As the data were acquired using questionnaires, a certain amount of bias cannot be excluded. This is especially true for the outcome parameter “time to upload or view,” which is only a subjective estimation but has been treated as a categorical variable. That is the main limitation of this study. For digital camera usage, in particular, an electronic measurement of these parameters was not feasible, and neither were such analyses incorporated in the app. However, any bias should influence both the data acquired from the digital camera and the smartphone app similarly, and we only aimed to analyze any differences found between them. Therefore, the evidence should not be relevantly impaired by these limitations. Another limitation is that smartphone photography can compete with digital cameras in regard to the standards and quality of small versions meant for small everyday tasks but not for scientific, educational, or other more challenging purposes requiring higher resolution and quality. Especially in light of this, another limitation is that usage in general was assessed, not the situations, the content, or the quality of the photographs. In this study, however, the sensor and resolution of the cameras were comparable on both devices.

Finally, no questioning or evaluation of the patients’ comfort and preference with both devices has been conducted.

### Conclusions

Specialized smartphone apps offer a secure, fast, and easy way to acquire medical photos and could possibly improve patient education and care in terms of wound management, in particular, while also offering medicolegal and economic benefits. Future studies should focus on a more objective assessment of differences and take factors such as patient comfort and preference, image resolution, and picture quality into consideration, as well as a larger sample size.

## Appendix

Multimedia Appendix 1 Questionnaire on the daily usage of a digital camera for photo documentation in everyday clinical practice.

Multimedia Appendix 2 Questionnaire on the daily usage of the mobile application “mRay Foto” for photo documentation in everyday clinical practice.

Multimedia Appendix 3 Questionnaire for the qualitative survey at study completion.

Multimedia Appendix 4 Standard Operating Procedure Medical Photography.

## Abbreviations

PACS: picture archiving and communication system

## Data Availability

The data sets used and analyzed during this study are available from the corresponding author on request.

## References

[1] O'Farrell P, Ferreira N (2016). Digital photography in orthopaedics: ethical considerations. SA Orthop J.

[2] Brandl D, Prantl L (2019). Fotodokumentation ästhetischer Behandlungen. Article in German. J Ästhet Chir.

[3] Arimany Manso J, Taberner Ferrer R, Pidevall I, Mascaró Ballester J, Martin-Fumadó C (2020). Use of photography in dermatology: ethical and legal implications. Actas Dermo-Sifiliográficas (English Edition).

[4] Creighton S, Alderson J, Brown S, Minto CL (2008). Medical photography: ethics, consent and the intersex patient. BJU International.

[5] Grassberger M, Verhoff MA (2013). Klinisch-forensische Fotodokumentation. Klinisch-forensische Medizin.

[6] Eichhorn C, Nagel E (2009). Fotodokumentation. Article in German. Praev Gesundheitsf.

[7] Karim RB, Hage JJ, Ahmed AKJ, de Wit FS, van de Sandt MM, Daemen A (2002). Digital photography as a means of enhancing interconsultant communication in oncological cutaneous surgery. Ann Plast Surg.

[8] DeLange GS, Diana M (1999). 35 mm film vs. digital photography for patient documentation: is it time to change?. Ann Plast Surg.

[9] Galdino GM, Swier P, Manson PN, Vander Kolk CA (2000). Converting to digital photography: a model for a large group or academic practice. Plast Reconstr Surg.

[10] de Almeida Geraldino R, de Lucas Rezende LVM, da-Silva CQ, Almeida JCF (2017). Remote diagnosis of traumatic dental injuries using digital photographs captured via a mobile phone. Dent Traumatol.

[11] Ndong A, Diallo AC, Faye M, Ndiaye M, Diouf A, Faye PM, Niasse A, Thiam JA, Sarr ISS, Seye Y, Gueye ML, Thiam O, Seck M, Touré AO, Cissé M, Ka O, Dieng M (2019). Clinical photography in surgery: knowledge, attitudes and practices in Dakar. Int J Surg Sci.

[12] Solan MC, Calder JD, Gibbons CE, Ricketts DM (2001). Photographic wound documentation after open fracture. Injury.

[13] Wade FA, Oliver CW, McBride K (2000). Digital imaging in trauma and orthopaedic surgery: is it worth it?. J Bone Jt Surg Br Vol.

[14] Regulation G (2016). Regulation (EU) 2016/679 of the European Parliament and of the council of 27 april 2016. Official Journal of the European Union.

[15] Roguljić M, Šimunović D, Peričić TP, Viđak M, Utrobičić A, Marušić M, Marušić A (2022). Publishing identifiable patient photographs in scientific journals: scoping review of policies and practices. J Med Internet Res.

[16] Cheung A, Al-Ausi M, Hathorn I, Hyam J, Jaye P (2005). Patients' attitudes toward medical photography in the emergency department. Emerg Med J.

[17] Harting MT, DeWees JM, Vela KM, Khirallah RT (2015). Medical photography: current technology, evolving issues and legal perspectives. Int J Clin Pract.

[18] Hsieh C, Yun D, Bhatia AC, Hsu JT, de Luzuriaga AMR (2015). Patient perception on the usage of smartphones for medical photography and for reference in dermatology. Dermatol Surg.

[19] Lau CK, Schumacher HHA, Irwin MS (2010). Patients' perception of medical photography. J Plast Reconstr Aesthet Surg.

[20] Accetta JL, Schoenfeld J, Bitar C, Murina A (2020). Smartphones in dermatology: acceptance of smartphone photography by the informed patient. Dermatol Surg.

[21] Jordan MC, Jovic S, Gilbert F, Kunz A, Ertl M, Strobl U, Jakubietz RG, Jakubietz MG, Meffert RH, Fuchs KF (2021). Smartphone-based photographic wound documentation improves the quality of medical accounting in orthopedic and plastic surgery. Article in German. Unfallchirurg.

[22] Nair AG, Potdar NA, Dadia S, Aulakh S, Ali MJ, Shinde CA (2019). Patient perceptions regarding the use of smart devices for medical photography: results of a patient-based survey. Int Ophthalmol.

[23] Bronsard N, Sicard BC, Amoretti N, Rottier H, Ertz P, de Peretti F (2015). Interest of including trauma photography in the picture archiving and communication system of a teaching hospital. Orthop Traumatol Surg Res.

[24] Schröder G (2019). Bei Wunddokumentation nicht verunsichern lassen. Article in German. Heilberufe.

[25] Sanger PC, Simianu VV, Gaskill CE, Armstrong CAL, Hartzler AL, Lordon RJ, Lober WB, Evans HL (2017). Diagnosing surgical site infection using wound photography: a scenario-based study. J Am Coll Surg.

[26] Bouillon B, Marzi I (2018). The updated German "Polytrauma—Guideline": an extensive literature evaluation and treatment recommendation for the care of the critically injured patient. Eur J Trauma Emerg Surg.

[27] Friesen MR, Hamel C, McLeod RD (2013). A mHealth application for chronic wound care: findings of a user trial. Int J Environ Res Public Health.

[28] Verhoff MR, Kettner M, Lászik A, Ramsthaler F (2012). Digital photo documentation of forensically relevant injuries as part of the clinical first response protocol. Dtsch Arztebl Int.

[29] Esser P (2019). Fotodokumentation: Gebühren-, Technik- oder keine Leistung? Article in German. Der Freie Zahnarzt.

[30] Bryson D (1999). Operating theatre photography for personal injury cases. J Audiov Media Med.

[31] de Carvalho RMF, Mazzer N, Barbieri CH (2012). Analysis of the reliability and reproducibility of goniometry compared to hand photogrammetry. Acta Ortop Bras.

[32] Naylor JM, Ko V, Adie S, Gaskin C, Walker R, Harris IA, Mittal R (2011). Validity and reliability of using photography for measuring knee range of motion: a methodological study. BMC Musculoskelet Disord.

[33] Li MKH, Howard DP, King R (2019). "A picture tells a thousand words" smartphone-based secure clinical image transfer improves compliance in open fracture management. Injury.

[34] Kazemi T, Lee KC, Bercovitch L (2019). Just a quick pic: ethics of medical photography. J Am Acad Dermatol.

[35] Archibald DJ, Carlson ML, Friedman O (2010). Pitfalls of nonstandardized photography. Facial Plast Surg Clin North Am.

[36] de Meijer PPG, Karlsson J, LaPrade RF, Verhaar JAN, Wijdicks CA (2012). A guideline to medical photography: a perspective on digital photography in an orthopaedic setting. Knee Surg Sports Traumatol Arthrosc.

[37] Uzun M, Bülbül M, Toker S, Beksaç B, Kara A (2014). Medical photography: principles for orthopedics. J Orthop Surg Res.

[38] Kim SH, Sobez LM, Spiro JE, Curta A, Ceelen F, Kampmann E, Goepfert M, Bodensohn R, Meinel FG, Sommer WH, Sommer NN, Galiè F (2020). Structured reporting has the potential to reduce reporting times of dual-energy x-ray absorptiometry exams. BMC Musculoskelet Disord.

[39] Körber A, Rietkötter J, Grabbe S, Dissemond J (2006). Three-dimensional documentation of wound healing: first results of a new objective method for measurement. J Dtsch Dermatol Ges.

[40] Wang S, Zhang Q, Huang W, Tian H, Hu J, Cheng Y, Peng Y (2018). A new smart mobile system for chronic wound care management. IEEE Access.

