# Supplementary material for: Improving Medical Photography in a Level 1 Trauma Center by Implementing a Specialized Smartphone-Based App in Comparison to the Usage of Digital Cameras: Prospective Panel Study
Source: JMIR Form Res. 2024 Jan 25;8:e47572. doi: 10.2196/47572 (PMC10853857; doi:10.2196/47572)
Supplement: Multimedia Appendix 1 [file formative_v8i1e47572_app1.pdf]

*Study coordinator: Dr. Sven Vetter, BG Trauma Center Ludwigshafen, Tel.: +49 (6 21) 68 10 – 8823, sven.vetter@bgu-ludwigshafen.de*

| **Questionnaire on the daily usage of a digital camera for photo documentation in everyday clinical practice** | |
|---|---|
| Your identifier: | ______________________________ |
| Date: | ☐☐.☐☐.2020 |
| 1. How relevant is photo-documentation in your daily clinical practice? | Not at all ○ ○ ○ ○ ○ Very |
| 2. Which clinical conditions do you document in your daily clinical routine? (Multiple selection possible!) | ○ Soft tissues<br>○ Wounds<br>○ Deformities<br>○ Range of motions (ROM)<br>○ Others:<br>______________________________ |
| 3. What is the purpose of photo documentation of clinical conditions in your case? | ○ Legal requirements<br>○ Improving therapy<br>○ Preoperative planning<br>○ Postoperative control<br>○ Consultation<br>○ Others:<br>______________________________ |
| 4. How often do you document clinical findings photographically per day? | approx. ☐ times per day |
| 4.1. How often per week did you not have your digital camera at hand? | approx. ☐ times |
| 5. How long does it take for the images to be archived in the clinical database (from capture to upload)? | approx. ☐ seconds<br>approx. ☐ minutes<br>approx. ☐ hours<br>approx. ☐ days |
| 5.1. What were the reasons for a potential delay? | ○ Technical issues<br>○ Distance to the closest available workstation<br>○ Organisational issues<br>○ No delay<br>○ Others:<br>______________________________ |

| | |
|---|---|
| 6. How much time does the technical archiving process take in total? | ○ < 10 seconds<br>○ 10-30 seconds<br>○ 30-60 seconds<br>○ 1-5 minutes<br>○ > 5 minutes |
| 7. How often do you use the digital camera per day to demonstrate the photo findings to a work colleague (second opinion/consult)? | approx. ☐ times |
| 7.1. How often per week do you not have your digital camera at hand? | approx. ☐ times |
| 8. How long does it take you to view the images of other work colleagues in the clinical database at a PC workstation (from request to viewing)? | ○ < 10 seconds<br>○ 10-30 seconds<br>○ 30-60 seconds<br>○ 1-5 minutes<br>○ > 5 minutes |
| 8.1. What were the reasons for a potential delay? | ○ Technical issues<br>○ Distance to the closest available workstation<br>○ Organisational issues<br>○ No delay<br>○ Others: ____________ |
| 9. How often were you unable to use the digital camera for technical reasons? | approx. ☐ times |
