# Supplementary material for: Improving Medical Photography in a Level 1 Trauma Center by Implementing a Specialized Smartphone-Based App in Comparison to the Usage of Digital Cameras: Prospective Panel Study
Source: JMIR Form Res. 2024 Jan 25;8:e47572. doi: 10.2196/47572 (PMC10853857; doi:10.2196/47572)
Supplement: Multimedia Appendix 2 [file formative_v8i1e47572_app2.pdf]

Study coordinator: Dr. Sven Vetter, BG Trauma Center Ludwigshafen, Tel.: +49 (6 21) 68 10 – 8823, sven.vetter@bgu-ludwigshafen.de

**Questionnaire on the daily usage of the mobile application „mRay Foto" for photo documentation in everyday clinical practice**	
Your identifier:	______________________________
Date:	☐☐.☐☐.2020
1. How relevant is photo-documentation in your daily clinical practice?	Not at all ○ ○ ○ ○ ○ Very
2. Which clinical conditions do you document in your daily clinical routine? (Multiple selection possible!)	○ Soft tissues<br>○ Wounds<br>○ Deformities<br>○ Range of motions (ROM)<br>○ Others:<br>______________________________
3. What is the purpose of photo documentation of clinical conditions in your case?	○ Legal requirements<br>○ Improving therapy<br>○ Preoperative planning<br>○ Postoperative control<br>○ Consultation<br>○ Others:<br>______________________________
4. How often do you document clinical findings photographically per day?	approx. ☐ times per day
4.1. How often per week did you not have the smartphone with mRay photo at hand?	approx. ☐ times
5. How long does it take for the images to be archived in the clinical database (from capture to upload)?	approx. ☐ seconds<br>approx. ☐ minutes<br>approx. ☐ hours<br>approx. ☐ days
5.1. What were the reasons for a potential delay?	○ Technical issues<br>○ Distance to the closest available workstation<br>○ Organisational issues<br>○ No delay<br>○ Others:<br>______________________________

6. How much time does the technical archiving process take in total?	○ < 10 seconds<br>○ 10-30 seconds<br>○ 30-60 seconds<br>○ 1-5 minutes<br>○ > 5 minutes
7. How often do you use the smartphone with mRay photo per day to demonstrate the photo findings to a work colleague (second opinion/consult)?	approx. ☐ times
7.1. How often per week do you not have the smartphone with mRay photo at hand?	approx. ☐ times
8. How long does it take you to view the images of other work colleagues in the clinical database using mRay Photo (from request to view)?	○ < 10 seconds<br>○ 10-30 seconds<br>○ 30-60 seconds<br>○ 1-5 minutes<br>○ > 5 minutes
8.1. What were the reasons for a potential delay?	○ Technical issues<br>○ Distance to the closest available workstation<br>○ Organisational issues<br>○ No delay<br>○ Others: ______________________
9. How often were you unable to use mRay Foto for technical reasons? (e.g. restricted WLAN, software error, network failure, etc.)	approx. ☐ times
