# Supplementary material for: Improving Medical Photography in a Level 1 Trauma Center by Implementing a Specialized Smartphone-Based App in Comparison to the Usage of Digital Cameras: Prospective Panel Study
Source: JMIR Form Res. 2024 Jan 25;8:e47572. doi: 10.2196/47572 (PMC10853857; doi:10.2196/47572)
Supplement: Multimedia Appendix 3 [file formative_v8i1e47572_app3.pdf]

„mRay Foto“ – Study
Questionnaire for the qualitative survey at study completion

This questionnaire is used for the final recording of qualitative variables, such as general satisfaction. Except for a few fields, no free text is expected, that is, the questionnaire should take less than 10 minutes to complete.
Thank you for your participation! If you have any questions, please contact Dr. Vetter.

Your identifier:	____________________
Date:	☐☐.☐☐.2020
1. How long have you been working as a resident?	approx. ☐ years
2. Are you using smartphones and/or tablets/computers privately?	○ No<br>○ Yes
2.1. If so: How often do you use them privately?	Rarely ○ ○ ○ ○ ○ Very often
3. What was your overall impression of the app?	Not usable ○ ○ ○ ○ ○ Very good
4. Where do you see the main benefit of the app when used in the inpatient setting of trauma surgery? (Multiple selection possible!)	○ I can save time in my daily work<br>○ The quality of treatment increases because communication is easier<br>○ Working is easier and more comfortable<br>○ Has no benefit
5. How much time could you save by using the app?	No difference ○ ○ ○ ○ ○ Viel mehr
6. Did you look more often at photo data by using the app?	No difference ○ ○ ○ ○ ○ More frequently
7. Has the app improved communication with colleagues?	No difference ○ ○ ○ ○ ○ Much easier
8. How do you rate the usage of the app overall?	Unsatisfactory ○ ○ ○ ○ ○ Very good
8.1. How do you rate the responsiveness of the app?	Unsatisfactory ○ ○ ○ ○ ○ Very good
8.2. How do you rate the user interface of the app?	Unsatisfactory ○ ○ ○ ○ ○ Very good
8.3. How do you rate the clarity of the user interface?	Unsatisfactory ○ ○ ○ ○ ○ Very good
8.4. How do you rate the intuitivity of the user interface?	Unsatisfactory ○ ○ ○ ○ ○ Very good
9. Have the available functions of the app been sufficient for you to carry out your image evaluation?	○ Yes<br>○ No

10. Which functions would you still like to see in the app? (Multiple selection possible!)	○ Automated measurement of wounds<br>○ Automated assessment of soft tissues/wound conditions<br>○ Commenting on photo documentation<br>○ Others: ______________________
11. Do you have any other comments or suggestions for improvement?	______________________
