# Supplementary material for: Improving Medical Photography in a Level 1 Trauma Center by Implementing a Specialized Smartphone-Based App in Comparison to the Usage of Digital Cameras: Prospective Panel Study
Source: JMIR Form Res. 2024 Jan 25;8:e47572. doi: 10.2196/47572 (PMC10853857; doi:10.2196/47572)
Supplement: Multimedia Appendix 4 [file formative_v8i1e47572_app4.docx]

**Standard Operating Procedure Medical Photography**

**1. Objective and Scope of the SOP**

- Standardized photographic representation of decubitus ulcers, wounds, and wound progression
- Unambiguous linkage of photographic data to the patient's medical record
- Image quality facilitating an accurate and comprehensive wound assessment

**2. Applicability**

- All staff across all units and wards involved in patient care.

**3. Workflow Steps**

- Definition
- Nursing Interventions a. Pre-Operative Preparation b. Execution
- Special Considerations / Complications
- Post-Operative Procedure

**4. Description of Workflow Steps**

Note: Photographic documentation is complementary to, but not a substitute for, written wound descriptions.

- - **Pre-Operative Preparation**
    - Inform the patient about the rationale and objectives of the photographic documentation
    - Obtain verbal consent from the patient and document it in the Interdisciplinary Admission Form.
    - If mandated, engage the specialized photographic department.
  - **Execution**
    - Prior to capturing the image, cleanse the wound and place a sterile cloth underneath it.
    - When feasible, photograph in a room with the light source situated behind the photographer.
    - Employ flash photography whenever possible.
    - Orient photographs towards the head of the patient (the top margin of the image should point towards the patient's head).
    - If required, document the patient's positioning prior to the wound imaging.
    - Maintain a consistent distance between the wound and the camera lens.
    - Avoid angled photography of the wound.
    - Ensure high-quality, sharp images; delete any blurred pictures.
    - The wound should occupy at least one-third of the image and be clearly discernible.
    - To calibrate wound dimensions, include a wound ruler in the photograph, annotated with wound location.
    - Include photo number, patient's name, birth date, and staff identification mark on the wound ruler in the photograph.
- **Frequency**
  - To facilitate longitudinal comparison, capture images at the onset, upon notable wound changes, every two weeks at most, or prior to discharge.
- **Image Integration into KIS System**
  - This involves careful and responsible data handling, particularly in terms of data protection and security. Any attempts to misuse or input unauthorized data are strictly prohibited.
  - Complete tagging of the wound type, location, etc. must be included.
  - Follow guidelines in the KIS manual for image integration.
- **Special Considerations**
  - Photographic documentation legally constitutes an intrusion into protected personal rights.
  - Obtain consent from a legally authorized representative if necessary.
  - Image documentation is subject to data protection laws.
  - Use of images for internal/external educational purposes, symposia, etc., requires special written consent from the patient.

**5. Quality Indicators**

The following metrics will be evaluated as part of quality assurance:

- Completed photographic documentation, tagging, accurate labeling, temporal sequence of images, and their quality.
- Setting of quality indicators at the nursing station.
- Presence of informed consent.
